# Arrival times by Recurrent Neural Network for induced seismic events from a permanent network

**DOI:** 10.3389/fdata.2023.1174478

**Published:** 2023-08-04

**Authors:** Petr Kolar, Umair bin Waheed, Leo Eisner, Petr Matousek

**Affiliations:** ^1^Institute of Geophysics of the Czech Academy of Sciences, Prague, Czechia; ^2^Department of Geosciences, King Fahd University of Petroleum and Minerals, Dhahran, Saudi Arabia; ^3^Seismik s.r.o., Prague, Czechia

**Keywords:** Recurrent Neural Network, automatic arrival time detection, location, magnitude, hydraulic fracturing, induced seismicity, traffic light system

## Abstract

We have developed a Recurrent Neural Network (RNN)-based phase picker for data obtained from a local seismic monitoring array specifically designated for induced seismicity analysis. The proposed algorithm was rigorously tested using real-world data from a network encompassing nine three-component stations. The algorithm is designed for multiple monitoring of repeated injection within the permanent array. For such an array, the RNN is initially trained on a foundational dataset, enabling the trained algorithm to accurately identify other induced events even if they occur in different regions of the array. Our RNN-based phase picker achieved an accuracy exceeding 80% for arrival time picking when compared to precise manual picking techniques. However, the event locations (based on the arrival picking) had to be further constrained to avoid false arrival picks. By utilizing these refined arrival times, we were able to locate seismic events and assess their magnitudes. The magnitudes of events processed automatically exhibited a discrepancy of up to 0.3 when juxtaposed with those derived from manual processing. Importantly, the efficacy of our results remains consistent irrespective of the specific training dataset employed, provided that the dataset originates from within the network.

## Introduction

**Figure 1:**
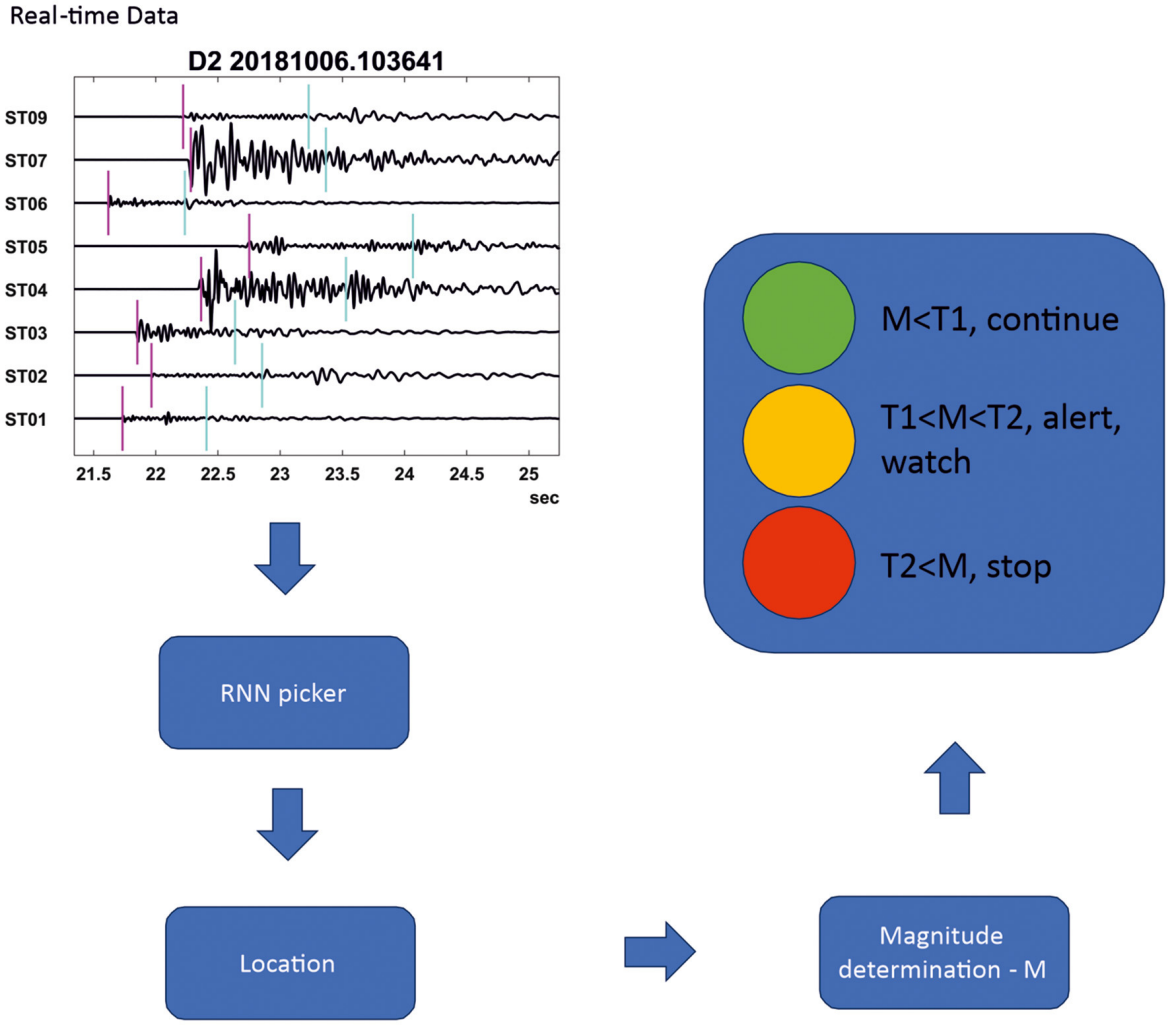
Illustration of use of RNN picker in the real-time processing of the traffic light system designed to mitigate large induced seismicity.

Seismicity induced by underground injections has been known for more than five decades (Raleigh et al., 1976), although recent development of unconventional resources has raised the issue of induced seismicity to a very high level of interest (Ellsworth, 2013). The most common mitigation strategy for adverse effects of induced seismicity is to apply the so called Traffic Light Systems—TLS (Häring et al., 2008; Verdon and Bommer, 2021). TLS requires rapid detection, location, and magnitude determination of induced seismic events in the vicinity of the hydraulic injection operations. Given that these injections typically operate on a continuous basis, seismic data processing must also be conducted continuously, typically using specialized surface monitoring arrays (Duncan Peter and Eisner, 2010). Figure 1 shows possible placement of an automated picker in the traffic light system illustrating the need for automation and processing in the real-time.

Large surface arrays detect and locate induced seismicity through the stacking of seismic signals. In contrast, smaller and more cost-effective arrays, comprised of up to tens of receivers, employ conventional short-term average/long-term average (STA/LTA) detection and phase picking techniques for localization. To facilitate automated processing from such smaller arrays, neural network-based location algorithms are often incorporated, allowing for continuous and rapid data processing. Such arrays continuously generate vast amounts of data, which are impractical to process manually. As a result, automated processing is typically employed. Several approaches exist for detecting seismicity, with the most prevalent method being the STA/LTA technique. This approach involves the detection of signals arriving at multiple stations, as measured by the ratio of short-term average to long-term average seismic energy (Allen, 1982). The STA/LTA method offers detection capabilities only, and it is susceptible to false detections depending on the chosen thresholds, and thus often necessitates manual intervention.

A novel methodology that integrates both detection and location capabilities leverages the Deep Neural Network (DNN) approach, originally developed for image classification tasks. One of the first efficient implementations of DNN was the Convolutional Neural Network (CNN) known as AlexNet (Krizhevsky et al., 2012), which rapidly gained popularity and was applied across various fields, including seismology (Mousavi and Beroza, 2022). The CNN approach has been applied to diverse datasets, encompassing global earthquakes (e.g., Mousavi et al., 2020), local networks (e.g., Woollam et al., 2019), acoustic emission laboratory data (e.g., Ciaburro and Iannace, 2022), as well as real-time earthquake early warnings (e.g., Li et al., 2018), and source mechanism parameter estimation (e.g., Kuyuk and Susumu, 2018; van den Ende and Ampuero, 2020).

When applying the DNN approach to seismograms, there are generally two methodologies: (i) treating seismograms as images, utilizing a CNN (Convolutional Neural Network), or (ii) considering them as time series, an RNN (Recurrent Neural Network). The first approach, which is widely used in contemporary seismological analyses, simulates human interpretation of seismograms and is akin to the analysis of analog seismograms. In contrast, the latter approach treats digital seismograms as time series. Under the CNN methodology, the processed seismogram is classified as an image containing pixel values, and the conversion of the CNN output to onset position(s) is indirect. Conversely, with the RNN approach, probabilities (e.g., of onsets) are calculated for the entire investigated record, and their maxima can be easily identified. In this context, Kirschner et al. (2019) employed a Long Short-Term Memory (LSTM) network—a specialized type of RNN—for phase picking on a local earthquake dataset. Additionally, Mousavi et al. (2019) developed a CNN-RNN earthquake detector (CRED) that combines convolutional layers and bidirectional LSTM units within a residual structure for robust detection of microearthquakes. In this study, we utilize an RNN by treating seismograms as time series and implement the algorithm proposed by Kolár and Petružálek (2022). In this approach, the RNNs are directly trained to produce a time course of onset probabilities. By adopting this methodology, we aim to enhance the efficiency and accuracy of seismic event detection and location in comparison to traditional techniques. This RNN-based approach offers a promising alternative for seismological applications, particularly when handling large volumes of data and the need for rapid, automated processing.

The DNN applications in seismology has been recently reviewed and compared with other methods (e.g., Mousavi and Beroza, 2022; Anikiev et al., 2023). It can be generally stated that as the DNN/CNN/RNN methodology is new (about a decade), therefore its use in seismology is not fully developed resulting in no general consensus on the most appropriate methodology for seismic processing. We chose the RNN architecture because we wish to have a general method which can be applied in areas where training datasets might be limited (Kolár and Petružálek, 2022). The CNN or DNN method may perform better but are designed for networks picks with good training dataset. The mentioned size limitation of the processed data does not enable application of such sophisticated DNN architectures as, e.g., U-Net (Ronneberger et al., 2015), CubeNet (Chen and Li, 2022), Tailoring Net (Anikiev et al., 2023), or W-Net (Lee and Lee, 2022) in our case.

**Figure 2:**
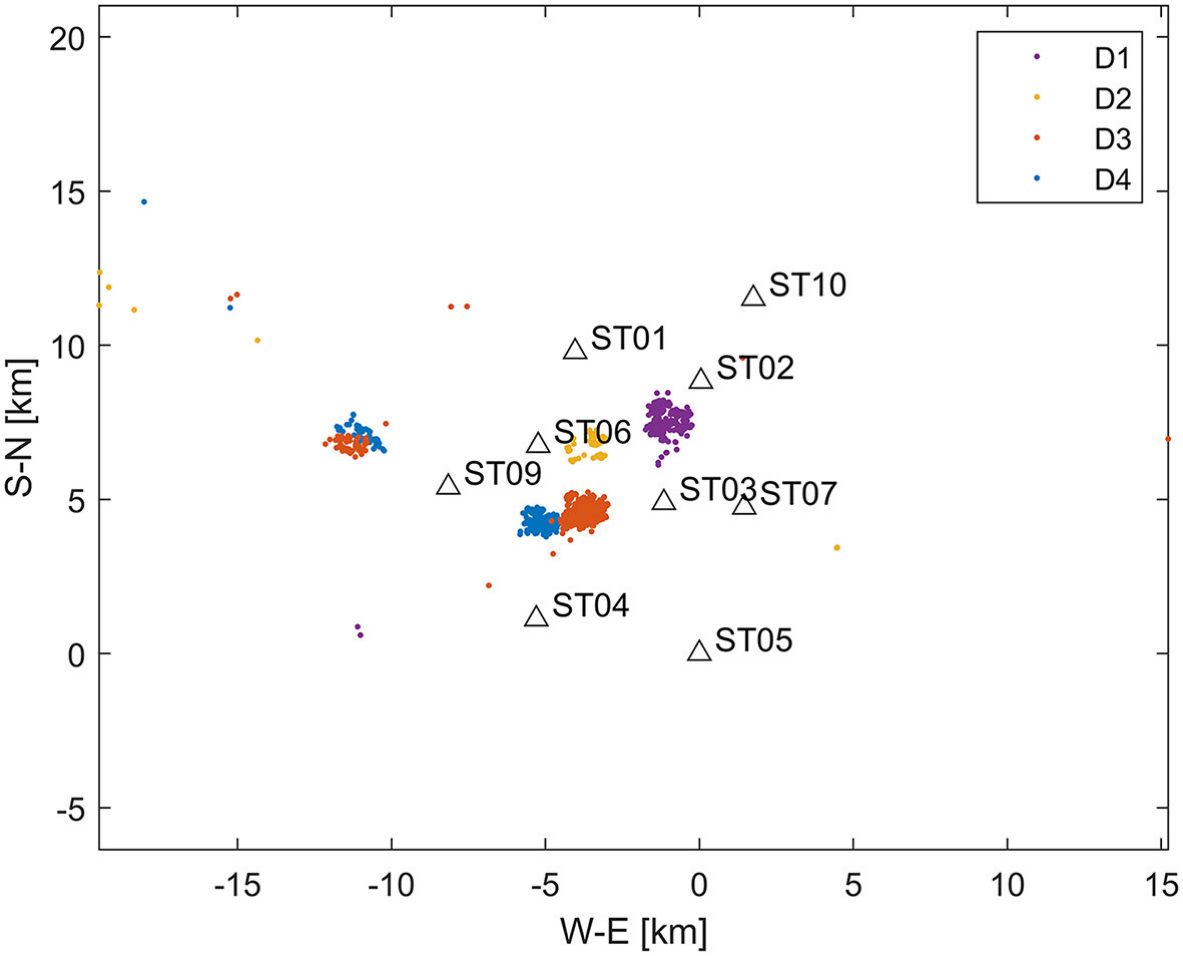
Map displaying station locations and epicenters of induced seismic events. Induced seismicity (datasets D1–D4) is detected at four wells (represented by color-coded dots), with nine network stations shown as triangles. The average depth of events is 3.4 km. It is important to note that while the induced seismic events are primarily clustered near stimulated wells, distant events (e.g., east of −15 km) are also observed, resulting from stimulations unrelated to the fracturing within the array. Station ST08 is not depicted, as it was an accelerometer with higher noise levels compared to geophones and was not utilized in this study.

**Figure 3:**
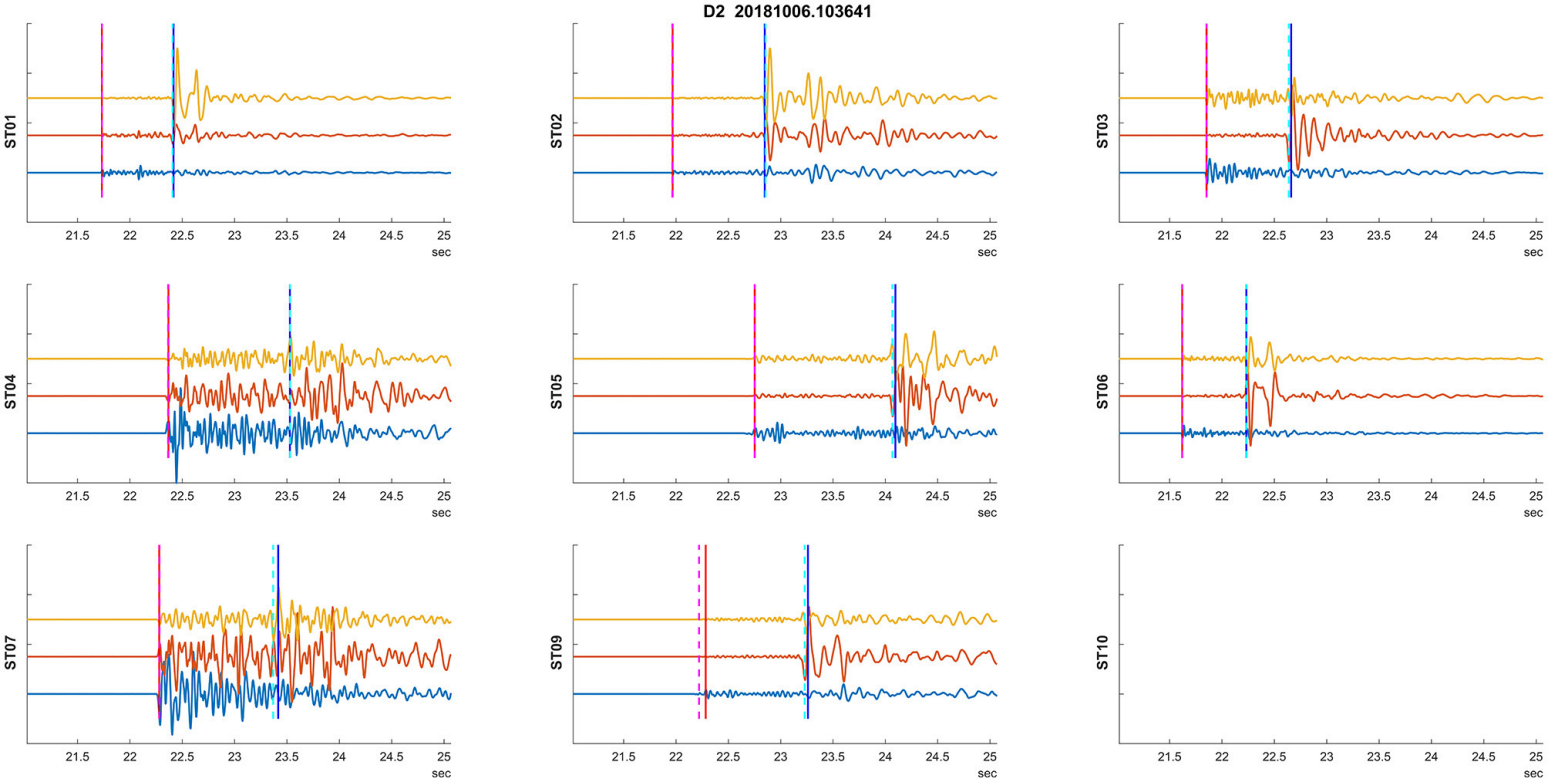
An example of a recorded induced seismic event. The plots depict three normalized components of particle velocity: vertical and two horizontal components (blue, red, and yellow) from 50 m deep borehole seismic stations. Manual arrival time detections are indicated by vertical dashed lines (red for P, cyan for S). Station ST10 was not recording during this event. The vertical lines signify automatic (RNN) picked arrival times.

We develop an algorithm tailored for long-term monitoring of induced seismicity, where a monitoring array is installed over a field in which operations are conducted across multiple wells (e.g., hydraulic fracturing of numerous wells) or during long-term injection processes (e.g., saltwater disposal, CO_2_ sequestration injection, and geothermal exploration). Monitoring arrays of this nature are typically deployed in shallow boreholes at depths of up to 200 m (Duncan Peter and Eisner, 2010) and are referred to as shallow buried arrays. Figure 2 illustrates the locations of induced seismic events stemming from four hydraulically stimulated wells, as well as the monitoring station geometry. Figure 3 presents the waveforms of a representative induced seismic event. The RNN methodology requires a learning dataset for training the neural network, which poses a challenge in induced seismicity monitoring, as there is typically no learning dataset available before the injection process begins. We propose an algorithm suited for a permanent network (e.g., Figure 2). In such a network, the first acquired dataset can serve as the learning dataset, enabling the trained RNN model to process the remaining datasets within the network in real-time. The numerical experiments in this study investigate the sufficiency of such learning and determine the expected accuracy. The initial dataset must be processed in real-time, either manually or through an alternative automated algorithm, and ideally post-processed with precise picking. A crucial consideration from the Traffic Light System (TLS) perspective is whether this approach yields reliable magnitudes for the induced seismic events. By developing an algorithm that addresses this challenge, we aim to enhance the efficiency and accuracy of induced seismicity monitoring in permanent networks.

Our proposed methodology offers a promising solution for long-term monitoring of induced seismicity, particularly in situations where multiple wells or ongoing injection processes are involved. By leveraging the RNN approach and incorporating an initial learning dataset, we strive to improve the real-time detection and analysis of induced seismic events, ultimately contributing to more effective mitigation strategies and risk assessment.

The next section describes the methodology including RNN architecture, followed by a section demonstrating the application on real data set (including RNN output post-processing). We conclude this study with a discussion of the methodology and the application before making final conclusions.

### Methodology

**Figure 4:**
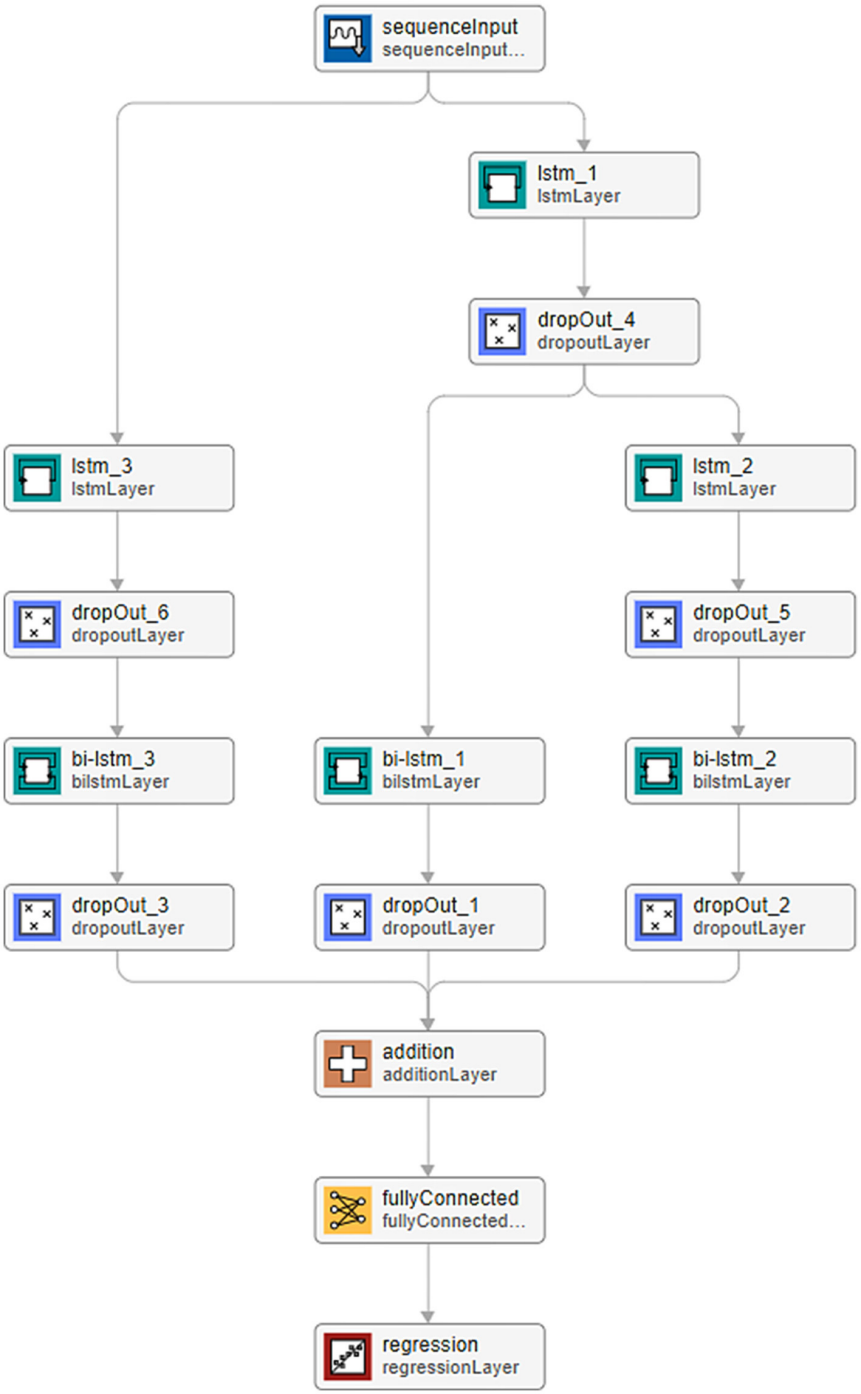
RNN Architecture. Each cell displays the RNN layer name (upper row: layer names are referenced in Table 1) and the CNN/RNN standard function used (lower row). A description of the individual layers used within the CNN/RNN formalism can be found in Witten and Frank (2016).

We process data from a nine three-component station network (Figures 2, 3) using a Recurrent Neural Network (RNN)^1^ to automatically detect the P and S wave onsets in the investigated seismograms. Our analysis assumes repeated monitoring of induced seismicity within the permanent network. The dataset comprises induced seismicity detected during the stimulation of four different wells. Our methodology assumes that the first set of induced events is processed independently, serving as a learning dataset for the RNN algorithm. Chen and Li (2022) and Zhu et al. (2022) employed an RNN comprising three parallel branches for earthquake arrival picking. We adopt a similar RNN architecture, as illustrated in Figure 4, with architecture details provided in Table 1.

**Table 1:** RNN parameters: the layers' names correspond to those used in Figure 4 [Drop_out layer value is identical in every application, the “|” symbol represents a connection, the individual layers are as described in Witten and Frank (2016)].

	**Sequence input: number of features: 3**	
**(Splitting)**		
|		Lstm_1 Num. of units: 21
|		Drop_out: Value: 0.15
|	(Splitting)	
Lstm_3 Num. of units: 21	|	Lstm_2 Num. of units: 14
Drop_out Value: 0.15	|	Drop_out Value: 0.15
Bi-lstm_3 Num. of units: 7	Bi_lstm_1 Num. of units: 7	Bi_lstm_2 Num. of units: 7
Drop_out Value: 0.15	Drop_out Value: 0.15	Drop_out Value: 0.15
**Addition**		
	Fully_connected Num. of classes: 3	
	Regression *(output)*	

A standard loss function is employed in the following form:


(1) $$\begin{array}{l} L\;=\;\frac{1}{2S}\;\sum_{i=1}^{S}\sum_{j=1}^{R}{\left(t_{ij}-y_{ij}\right)}^{2}\;, \end{array}$$

Where *t* is the target, *y* is the prediction time series, *S* is the time series length and *R* is the number of responses used. The learning efficiency is measured in terms of prediction accuracy percentage. The learning dataset is conventionally divided into three subsets: training, validation, and testing, with a standard ratio of 0.6/0.1/0.3 employed for the learning process. In this study, we examine alternative learning datasets (D1 and D4) to assess the robustness of the algorithm.

**Figure 5:**
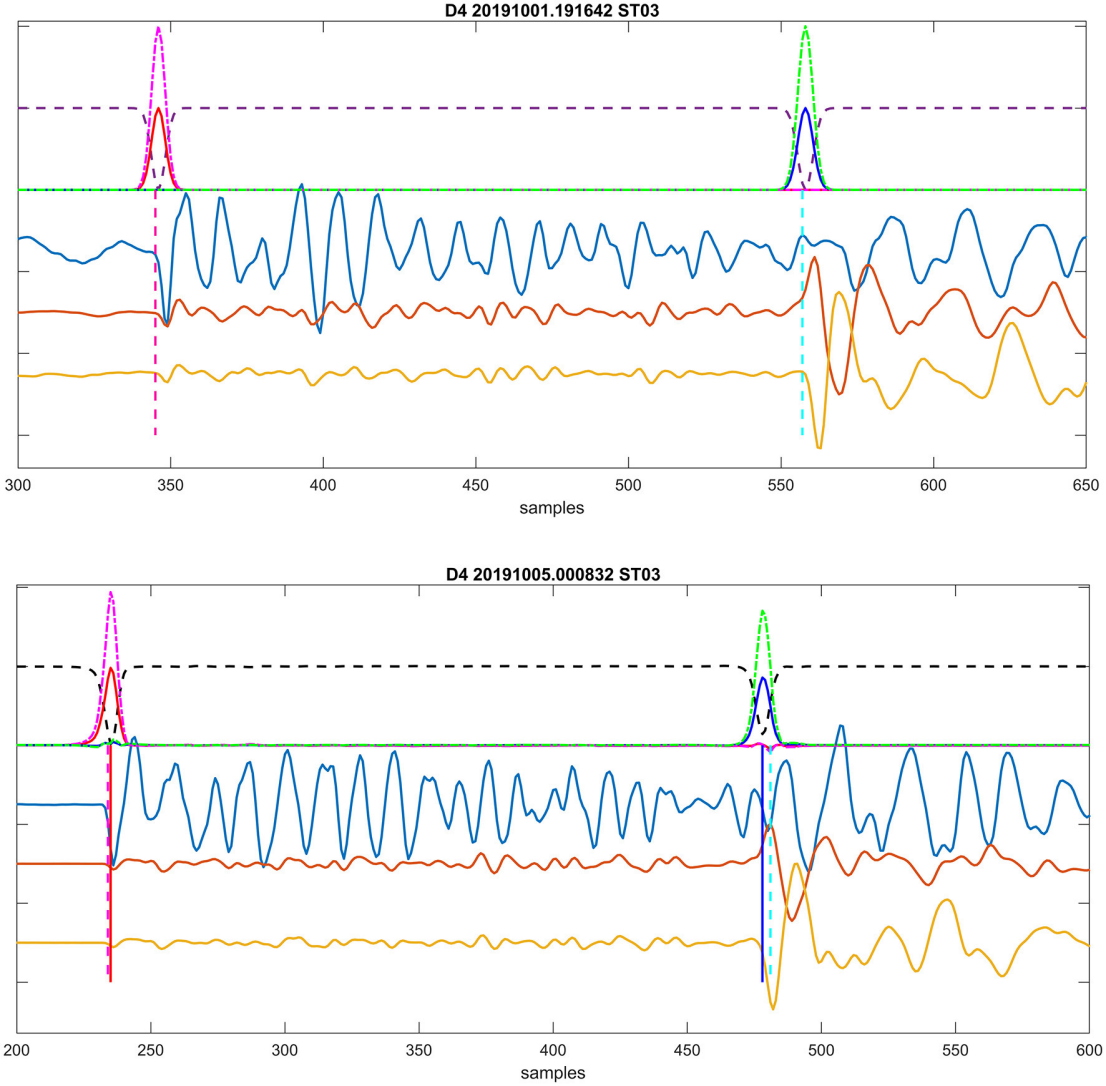
**(Top)** Target probabilities computed for an event from a training subset. Inputs include three components of particle velocity, shown as yellow, brown, and light blue traces corresponding to east, north, and vertical directions, respectively. Computed target probabilities are displayed as follows: *pP*, solid red line; *pS*, solid blue line; *pC*, dashed black; *pP*_*all*_, dashed-dotted magenta; and *pS*_*all*_, dashed-dotted green lines above. Positions of manual onset interpretations are indicated by vertical dashed lines: magenta for P and cyan for S. **(Bottom)** Example of successful prediction for an event from a testing subset. The lines' description is the same as in the top plot. Additionally, the automatically identified onsets' positions (corresponding to the maxima of predicted probabilities *pP*/*pS*_*all*_) are marked by full vertical lines: red for P and blue for S.

The RNN is employed in a sequence-to-sequence mode—see Chapter 2 in Jung (2022), with the three-component time interval of recorded unfiltered particle motion waveforms serving as input (the time interval length was set to 1,500 points in our case). These seismograms are randomly divided into training, validation, and testing subsets. The target function (the output) consists of three time series which represent probabilities: (i) the probability of occurrence P onset (*pP*)—a Gaussian curve centered on the most probable value, (ii) the S onset occurrence probability (*pS*) with a Gaussian curve, and (iii) a complementary value pC, constructed such that the total sum of these three functions (*pP*(*t*), *pSt*), *pC*(*t*) is one at every time sample. An example of target functions is depicted in Figure 5 (top), while Figure 5 (bottom) illustrates a successful prediction example. Similar target sequence functions were proposed by Woollam et al. (2019) and utilized in Kolár and Petružálek (2022).

It is important to note that although the sum of all three target sequences is defined as one at every time sample, these RNN outputs are independent functions. For the real data, their sum may not be exactly one. We employ a similar approach as used in Kolár and Petružálek (2022) to enhance detection capability, constructing P-wave and S-wave detection probabilities *pP*_*all*_(*t*) and *pS*_*all*_(*t*) as follows:


(2) $$\begin{array}{l} pP_{all}\left(t\right)=1+\;pP_{\;}\left(t\right)-pS\left(t\right)-pC\left(t\right), \end{array}$$

(3) $$\begin{array}{l} pS_{all}\left(t\right)=1+\;pS_{\;}\left(t\right)-pP\left(t\right)-pC\left(t\right). \end{array}$$

These values are evaluated from the three component RNN sequences output.

The maxima of *pP*_*all*_/*pS*_*all*_ are considered as arrival time detections if they reach a threshold of 0.05 for P waves or 0.1 for S waves, respectively. These threshold values were set through quick initial testing. An example of the automatic detection is shown in Figure 5 (bottom). In this case, the P-wave arrival times are practically identical (with a difference of 1 sample), while the automatic S-wave arrival times are slightly earlier (by 3 samples). An example of successful detection of arrival times for an event is then depicted in Figure 3.

**Figure 6:**
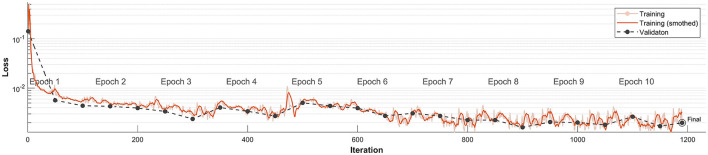
Loss function progression for training data set D1. The Loss function is represented by the solid orange line, while the corresponding validation value is the dashed black line. Note that the vertical ax is using logarithmic scale.

A crucial question during any CNN/RNN learning process is determining the proper (sufficient) number of iterations while avoiding potential overfitting. Figure 6 illustrates the CNN/RNN learning for dataset D1. We display the Loss function [defined in Equation (1)] obtained during the learning process; the function achieves stable values after the third epoch. This is generally considered an indication of a sufficiently learned network (i.e., enough iterations)—see Chapter 6.6 in Jung (2022). Note, that RMSE value (i.e., the standard RMS difference between prediction and target) which can also be used for such test, has in our case the same value (but for a multiplicative constant) as Loss function. The values of obtained Loss for the independent validation dataset also correspond fairly well to the values obtained from the training set. We view this as an indication that the network is not overtrained. Note that the loss function is plotted on semi logarithmic scale to visualize the values close to 0 of the function.

The main training on all datasets have used following controlling parameters: initial learning_rate = 0.022, gradient_threshold = 0.7 and batch_size = 20. These parameters were set empirically to provide reliable results in reasonable time. At the beginning of our study, we started with default values of these parameters, then we tried to optimize them by Bayesien hyperparameters optimization approach (Snoek et al., 2012). However, we did not observe strong dependency of results on these values.

### Application to real dataset

We have applied the described methodology to four datasets (referred to as D1–D4) acquired on a permanent monitoring network shown in Figure 2. The induced seismicity resulted from hydraulic fracturing at the depths between 2 and 3 km within the network. The events were located in a 1D layered velocity model derived from the well-log data and active seismic processing. The real-time monitoring of induced seismicity was supervised manually and required manual processing and verification of the preliminary automated processing which often failed. The recordings were later re-processed to achieved maximum consistency.

The real-time processed datasets were manually re-processed to obtain high-precision picks, locations, and magnitudes. The arrival times were manually picked at peaks or troughs of P- and S-wave arrivals to achieve maximum consistency between picked events and to minimize the impact of noise on weaker events. Furthermore, recorded waveforms were correlated with a template library and picks were refined based on the correlations to achieve greater consistency.

**Figure 7:**
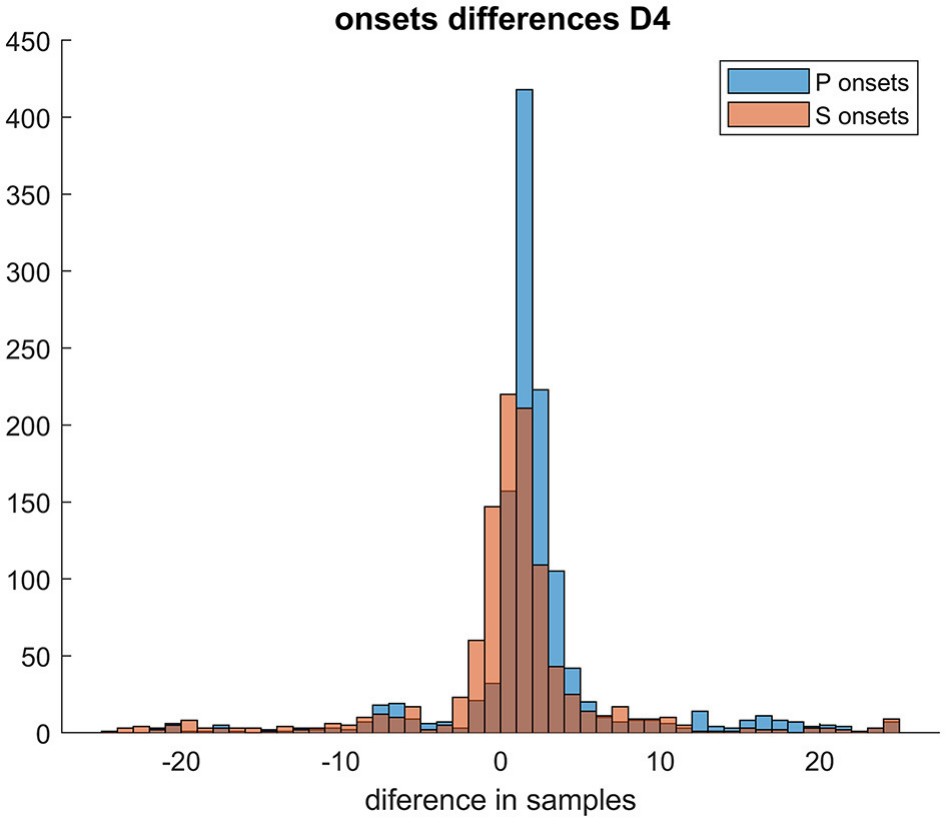
Histogram of differences between predicted and manually picked onsets for D4 dataset.

The number of samples in the Gaussian uncertainty for steps (i) and (ii) regarding P- and S-waves was chosen to be 5 and 6 samples, respectively. These values were determined based on the width of the observed signal. Table 2 summarizes the results of the automatic picking. We assess the arrival time accuracy (in %) of the RNN; the automated arrival time detection is considered successful if the difference between the automated arrival time (prediction) and manual time arrival (target) is < 20 samples, i.e., 0.08 s, with either learning dataset, over 80% of the automated picks were within 20 samples of the manual picks. This is also illustrated in an example of a histogram depicting the differences between automatic arrival times and manual arrival times, as shown in Figure 7. The chosen criterion for successful arrival time is relatively lenient, as the majority of prediction differences fall within the interval of ±5 samples (i.e., 0.02 s) for both P and S arrival times. In other words, our prediction would fulfill even stricter conditions for successful detection in the majority of processed events.

**Table 2:** Accuracy of automatic P and S onset detection for two bias datasets.

**Data set**	**Number of events/records**	**Trained on D1**		**Trained on D4**	
		**Accuracy**		**Accuracy**	
		**P**	**S**	**P**	**S**
D1	568/4,076	94.0%	96.1%	92.3%	76.4%
D2	82/532	83.3%	82.9%	86.1%	84.4%
D3	1,101/10,157	94.3%	96.5%	96.2%	94.2%
D4	603/4,466	88.6%	86.1%	92.8%	92.0%

Training datasets used are either D1 or D4.

However, arrival time-based locations can be sensitive to outliers. Therefore, to locate the seismic events with automated picks, we applied additional criteria for automatic arrival times: for each event and each station where both P and S-wave arrival times were detected, we check if *(tS-tP)* > *0.5 s*. If either of these criteria is met, both such picks are excluded from subsequent processing (location). For stations where only one of the arrival times (either P- or S-wave) is available, we use that arrival time. The criterion results in a condition that assumes local events occurring at depths >1 km. The number of excluded arrival times based on these criteria is listed in Table 3.

**Table 3:** Results of post-processing.

**Data set**	**Learned on D1/Num. of excluded seismograms tP > tS abs (tP-tS) < 0.5**	**Learned on D4/Num. of excluded seismograms tP > tS abs (tP-tS) < 0.5**
D1	8,129/7,935	8,120/7,398
D2	1,033/951	1,055/935
D3	20,227/19,724	20,165/19,517
D4	8,890/8,636	8,866/8,647

The number of automatic picks obtained directly from the network and the number of picks that meet post-processing criteria are given.

**Figure 8:**
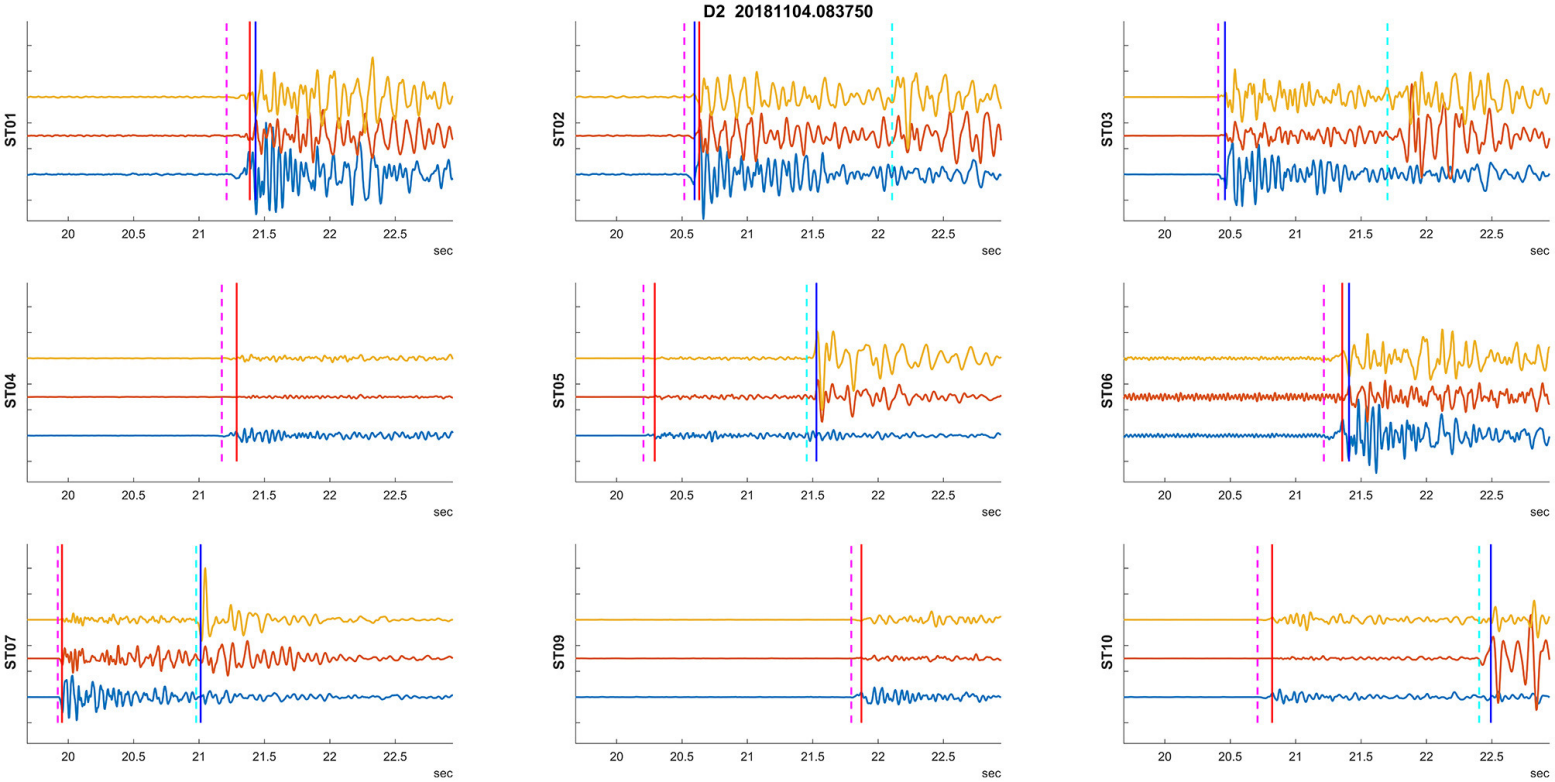
Example of a problematic auto picking. Plots utilize the same visualization of recorded waveforms and picks as in Figure 3.

The numerical experiment evaluated two types of training datasets to estimate the robustness of the methodology. Initially, we used D1 as the training dataset and investigated its application on D2–D4 sets. We also applied this learning to the training dataset itself (i.e., D1) to assess how the onset picking performs on the entire training dataset. An example of detected arrival times on seismograms for an event from dataset D2 is shown in Figure 3. Furthermore, to test the robustness of the methodology, we also used D4 as the training dataset and applied it to the same datasets. Figure 8 shows an example of the problematic automatic arrival time detection. This is one of the most problematic cases that had to be corrected by additional criteria on Tp and Ts arrival times. The criterion is not passed on stations ST01, ST02, ST03, and ST06. In contrast, an example of successful arrival time detection is given in Figure 3.

The automatically picked arrival times were used for events location to test whether they are good enough to provide real-time location and (mainly) magnitude information. These locations are compared with locations obtained from manual picks. For each location the normalized residuum *ResN* is given as:


(4) $$\begin{array}{l} ResN=\frac{\sqrt{\sum_{i=1}^{n}{\left(T_{i}^{A}-\;TT_{i}-T_{0}\right)}^{2}\;}\;}{\left(n-m\right)}, \end{array}$$

Where *T*${}_{i}^{A}$ is ith arrival time and *TT*_*i*_ is traveltime corresponding to the ith arrival time and *T*_0_ is origin time divided by number of degrees of freedom *(n-m)*, where *n* is number of arrival times and *m* is number of degrees of freedom, i.e., four for three coordinates of the hypocenter and one origin time.

A general overview of the whole data processing described above is briefly summarized below in algorithm in points form (including inputs and outputs):

1. Primary input: triggered time interval of raw data−3 components particle velocity at each station of the network.
2. time interval of 3 component seismograms (length 1,500 samples) around the expected onsets from individual stations (input for RNN).
3. RNN prediction of 3 component sequences of probabilities (length 1,500 samples) of P/S and complementary signal occurrence—output of RNN.
4. Determination of the time of P- and S-wave arrival time defined as time of the maximum values of predicted probabilities.
5. Arrival conditioning: excluding P- and S-wave arrival times if (tS-tP) < 0.5s.
6. Output: times of P/S onsets.
7. Post-processing: location evaluation *(to make the location more robust, the onsets with high residua are possibly excluded)*.
8. Post-processing (optional): magnitude Mw evaluation.

## Results

**Figure 9:**
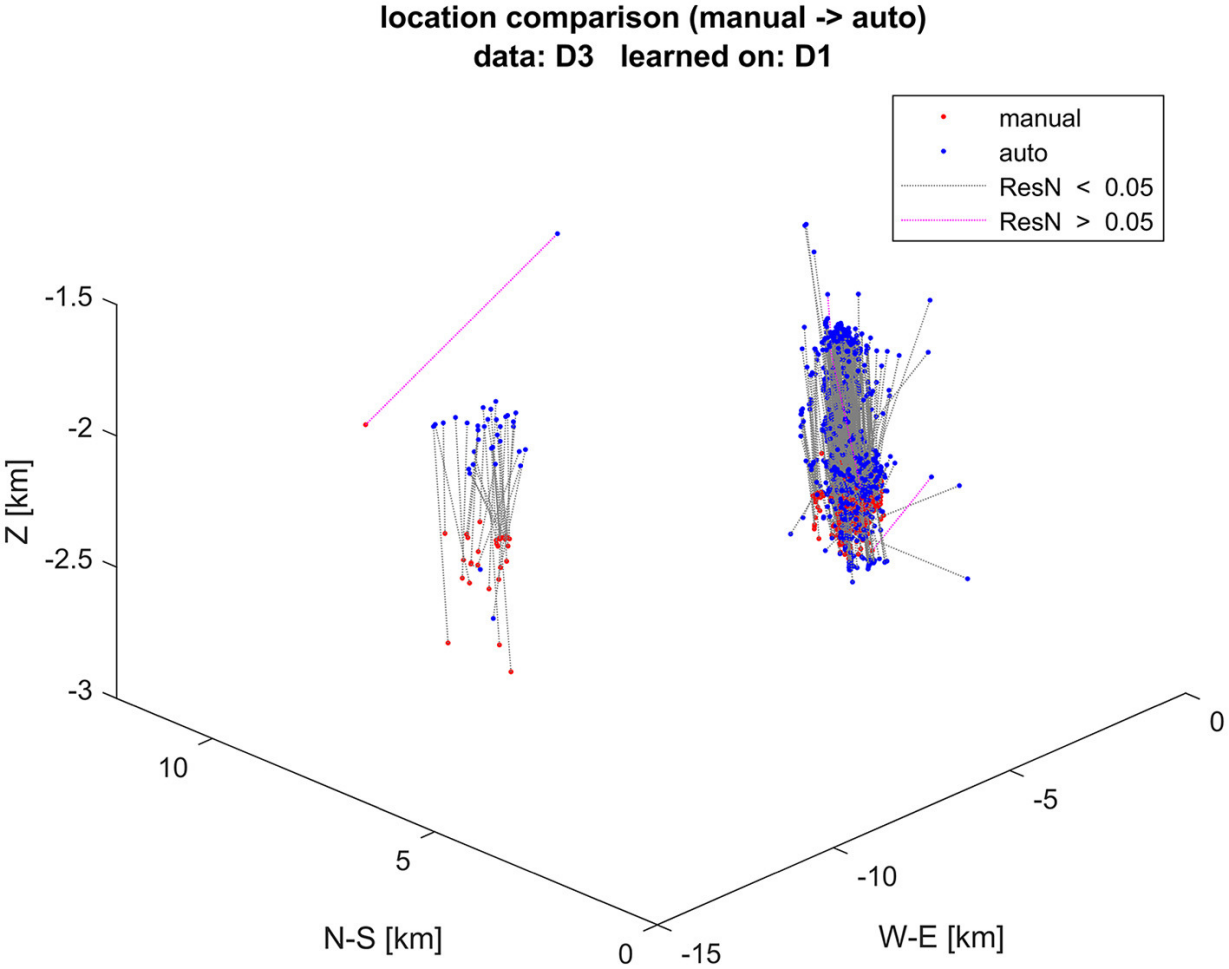
Event locations using manual (red dots) and automatic (blue dots) arrival times. Corresponding locations of the same event are connected by dotted lines. Events with normalized residuals ResN > 0.05 s are colored violet, while those with lower normalized residuals are colored black. The vertical axis is exaggerated.

Figure 9 shows comparison of event locations using manual and automatic onset detection with learning dataset D1, applied to dataset D3. Locations using automatic onset detection are more scattered. The locations determined by automatic picks are systematically shallower, with an average vertical difference of 240 m. The largest differences in locations correspond to the largest normalized residuals, as illustrated by the colors of the lines connecting the respective locations.

**Figure 10:**
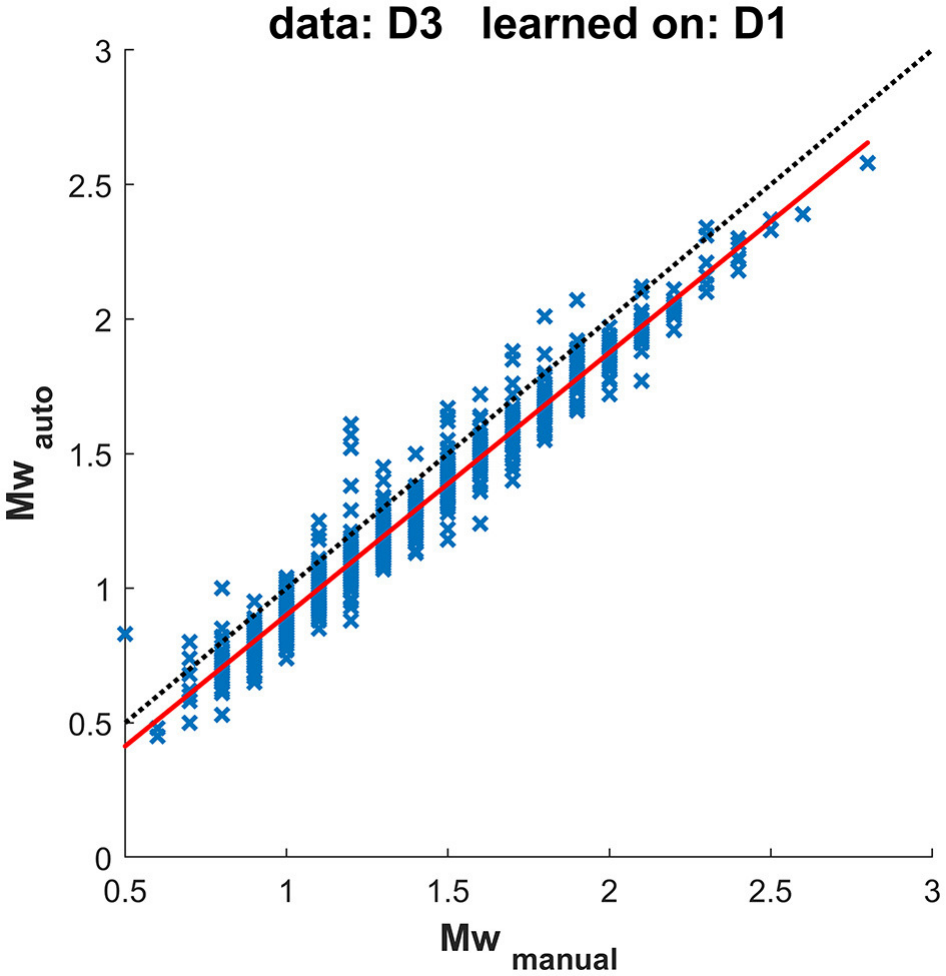
Magnitudes determined using manual and automatic picks, corresponding to the test in Figure 9. The dotted line represents a 1:1 relation, while the solid red line represents a linear interpolation through the observed values.

Figure 10 compares moment magnitudes (Mw) determined from manual and automatic onset detection and resulting locations for the same dataset, as shown in Figure 9. The moment magnitudes determined using automated arrival times are, on average, underestimated by −0.1, which is consistent with the shallower locations (locations closer to the receivers result in lower magnitudes).

The magnitude difference is small on average. However, TLS are dependent on a single observation of an event with magnitude exceeding certain threshold. Such events are with higher magnitude, Figure 10 shows that the differences for the events with *M*_*w*_> 1.5 are < 0.3 for majority of them and *all* < 0.5. Table 4 lists the maximum differences between magnitudes determined from manual and automatic processing for tested datasets using two different learning datasets; Table 5 gives the particular number of events within various moment magnitude differences level. Events with normalized location residuum smaller than 0.05 s do not underestimate the moment magnitude from the manual picks by more than 0.5. Events with magnitudes higher than *M*_*w*_1.5 and smaller normalized residuum than 0.05 s do not underestimate the manually determined magnitude by more than 0.3 for 98% of investigated events. Events with higher normalized residual may indicate some kind of failed automated processing and should be manually reviewed. the higher residual is usually observed for near-in-time or overlapping events (events with origin time difference < 4s had overlapping waveforms in this case study) where the automated arrivals usually fail. The automated arrival detection failure was indicated by high normalized residual value—therefore these events are not included in the presented results as they show only magnitude differences for events with low normalized residuals.

**Table 4:** Maximum differences between moment magnitude Mw using automatic and manual picks.

**Data-learned on**	**D1–D1**	**D2–D1**	**D3–D1**	**D4–D1**	**D2–D4**
Maximum of Mw (automatic picks)-Mw (manual picks) for ResN < 0.05 s	+0.11	+0.17	+0.41	+0.19	+0.26
Minimum of Mw (automatic picks)-Mw (manual picks) for ResN < 0.05 s	−0.46	−0.27	−0.36	−0.47	−0.27
Max observed deviation	−0.46	−0.67	+0.41	−0.47	−0.31

**Table 5:** Number of events with moment magnitude difference between automated and manual processing.

**Δ Mw value**	** < 0.2**	** < 0.3**	** < 0.4**	** < 0.5**	** < 0.6**	** < 0.7**	** < 0.8**
**Dataset/learning dataset**							
D1/D1	475	546	555	**559**			
D2/D1	75	80	80	80	80	**81**	
D3/D1	956	1,031	1,039	**1,040**			
D4/D1	574	588	591	**592**			
D2/D4	77	81	**82**				

The bold numbers represent all events detected in each dataset.

Our application of RNN methodology is simple. Allowing to use the trained algorithm on one network transfer the training to another similar network where no prior seismicity was previously observed. This application is going to be tested and may provide important advantage for induced seismicity for monitoring in areas where no prior seismicity occurred before.

### Computation cost

The computations were performed on a personal computer with 3.8 MHz Intel processor, 32 GB memory, two NVIDIA GeForce GTX 1070 external cards, Windows 10 system, on MATLAB 2022b platform. The learning process range from several tens of minutes to first hours (in dependency of number of considered records). The prediction for an individual event takes < 0.1 s, i.e., it could be considered as effectively on-line.

## Discussion

The input for the proposed DNN are individual seismograms (the seismograms are obtained on stations from a network), and the outputs of the algorithm are independently determined P or S arrival times of individual seismograms. We do not require or assume that the arrival times are mutually consistent, instead we assume the triggering algorithm which identified input for our processing triggered a time interval with one or more seismic events. This is both a drawback and an advantage. The advantage is that our trained DNN has potential to be directly used (i.e., without re-training) on the data from other comparable seismic networks. Testing of the assumption of direct transition of the trained network on other network data would be the first choice for future investigation in this field. The drawback of this methodology is that additional conditioning is required for locations as discussed in the above-described methodology.

Our tests indicate that the algorithm provides location and resulting automatic magnitude estimates with maximum observed error of 0.4 on the magnitude scale. Therefore, we believe that this algorithm can be used for real-time monitoring of hydraulic fracturing with the 0.4 magnitude margin. The algorithm can be also used for salt-water disposal monitoring as well as gas storage seismic monitoring.

In the numerical tests discussed in this study, we considered all events used by an operator to assess seismic hazard during hydraulic fracturing. Specifically, this dataset was complete down to events with Mw1.0. We have not excluded events with weaker signal-to-noise ratio. Should we need to apply this methodology to weaker induced seismic events we may need to study limits of this methodology for weaker microseismic events; however, this is not the target of this application where we investigate suitability for magnitude determination. Figure 10 does not show significant increase of magnitude error for lower magnitude events, indicating that we did not encounter significant effects of noise for events with Mw1.0 in our dataset. A more quantitative assessment of the signal-to-noise effects would require precise definition of signal and noise and set of much weaker events with manual picks for testing. Such set is not available in this dataset and if it was the manual picks would probably be unique and contain some level of error we would need to account for when comparing. Testing the limits on weak microseismic events is to be studied in the future tests.

## Conclusions

We have developed an automated method for detecting arrival times on triggered waveforms, suitable for repeated monitoring within a stationary monitoring array. This methodology is appropriate for real-time processing and provides arrival times that are suitable for determining event location and magnitude. The largest magnitude error in the tested datasets was < 0.5; however, 98% of the differences were < 0.3. The methodology is robust and does not appear to be dependent on a training dataset as long as it is within the monitoring array. The method either yields well-determined magnitudes or provides a warning for anomalous events (high residual). The Deep Neural Network (DNN) formalism has proven to be an efficient tool even for relatively small datasets (in our case, several hundred events from a moderate network) compared to larger sets used for EQ Transformer learning (Mousavi et al., 2020). Moderate datasets also imply a relatively simple architecture for the NN, as complex networks cannot be reliably trained on limited datasets. Despite this, our results are fairly acceptable, even for such data and NN architecture.

## Data availability statement

The data analyzed in this study is subject to the following licenses/restrictions: Confidential dataset. Requests to access these datasets should be directed to kol.pe@seznam.cz.

## Author contributions

PK developed the idea, trained the RNN, ran the tests, and prepared the draft. UW provided supervision to the work, improved the experiments, and revised the manuscript. LE supervised the project and participated in drafting and revising the text. PM helped with experiments and paper writeup. All authors contributed to the article and approved the submitted version.

## Footnotes

^1^CNN/RNN are general algorithms available in libraries of various programming languages; in our case, the computation was realized using MATLAB.
